# The Role of HLA-G in Human Papillomavirus Infections and Cervical Carcinogenesis

**DOI:** 10.3389/fimmu.2020.01349

**Published:** 2020-06-25

**Authors:** Hui-Hui Xu, Wei-Hua Yan, Aifen Lin

**Affiliations:** ^1^Medical Research Center, Taizhou Hospital of Zhejiang Province, Wenzhou Medical University, Linhai, China; ^2^Biological Resource Center, Taizhou Hospital of Zhejiang Province, Wenzhou Medical University, Linhai, China

**Keywords:** human leukocyte antigen G, human papillomavirus, viral infection, carcinogenesis, cervical cancer, immunotherapy

## Abstract

Human leukocyte antigen (HLA)-G, a non-classical HLA-class I molecule, has a low polymorphism frequency, restricted tissue distribution and immunoinhibitory property. HLA-G expression in tumor cells and cells chronically infected with virus may enable them to escape from host immune surveillance. It is well-known that the HLA-G molecule is a novel biomarker and potential therapeutic target that is relevant in various types of cancers, but its role in cervical cancer has not been fully explored. In this review, we aim to summarize and discuss the immunologic role of the HLA-G molecule in the context of HPV infections and the process of cervical cancer carcinogenesis. A better understanding of the potential impact of HLA-G on the clinical course of persistent HPV infections, cervical epithelial cell transformation, tumor growth, recurrence and metastasis is needed to identify a novel diagnostic/prognostic biomarker for cervical cancer, which is critical for cervical cancer risk screening. In addition, it is also necessary to identify HLA-G-driven immune mechanisms involved in the interactions between host and virus to explore novel immunotherapy strategies that target HLA-G/immunoglobulin-like transcript (ILT) immune checkpoints.

## Introduction

Cervical cancer ranks as the fourth most common female cancer worldwide, with an estimated 569,847 new cases and 311,365 deaths in 2018 (1). Persistent infection with high-risk human papillomavirus (hrHPV) is necessary but not sufficient to induce cervical cancer (2). Most HPV infections are transient and are cleared within months by host innate and adaptive immune responses (3). Failure to clear the virus leads to infection persistence, and only a minority of HPV-infected and transformed cells eventually avoid host immune surveillance, which leads to tumor growth and lymph node metastasis (4, 5). This host-dependent immunological status and HPV-induced immune escape are reflected in persistent infection and the subsequent progression of precancerous lesions to invasive cervical cancer, which indicates the complexity of host-virus interactions. Therefore, the roles of the immune system, not only in viral elimination but also in tumor antigen recognition, are extremely relevant in the process of cervical cancer carcinogenesis.

Accumulating evidence has supported the idea of a critical role for immunosuppressive mechanisms in promoting HPV-induced carcinogenesis, either by suppressing the capacity of the host to overcome HPV infection or by preventing the elimination of HPV-transformed epithelial cells (3–7). Human leucocyte antigen (HLA) complex is located on chromosome 6p21.3. Several HLA molecules with different functions can be broadly divided into classical HLA-class I (HLA-A, -B, -C), non-classical HLA-class I (HLA-E, -F, -G), classical HLA-class II (HLA-DR, -DQ, -DP), and classical HLA-class III (8). The HLA system influences the host immune response by mediating antigen presentation (9). HLA-G has been termed “non-classical” due to its low frequency of polymorphisms, restricted tissue distribution and immunoinhibitory properties, which are different from the properties of classical HLA-class I molecules (10, 11). It has become increasingly evident that the HLA-G molecule is involved in modulating both innate and adaptive immune responses and in promoting immune escape in various types of cancers (10–13) and infectious diseases (14–16). However, to date, the possibility that *HLA-G* gene polymorphisms and/or protein expression affecting HPV infection persistence and cervical cancer risk remains to be explored.

## Molecular Structure of Human Leukocyte Antigen-G

The *HLA-G* gene consists of eight exons, seven introns, a 5′upstream regulatory region (URR) that extends at least 1,400 bp from the initial ATG start codon, and a 3′untranslated region (UTR), with a total length of 6,000 bp (12, 17). It is widely accepted that the *HLA-G* primary transcript is alternatively spliced into seven mRNAs, which encode four membrane-bound (HLA-G1, -G2, -G3, -G4) and three soluble (HLA-G5, -G6, -G7) protein isoforms (18, 19). Each unique HLA-G isoform contains one to three extracellular globular domains (α1, α2, α3) encoded by exon 2, exon 3, and exon 4, whereas the presence of intronic sequences are variable (IMGT/HLA Database).

The overall structure of HLA-G1 and that of its soluble counterpart HLA-G5 is similar to the structure of the classical HLA-class I antigens, which contain a heavy chain non-covalently bound to β*2*-microglobulin (β*2*m) (18). Peptide is bound in the antigen-binding cleft formed by the α1 and α2 domains (11, 20), whereas the α3 domain can bind co-receptors such as CD8 (21). Both HLA-G1 and HLA-G5 isoforms can also exist as β*2*m-free antigens (22). Other HLA-G isoforms lacking one or two extracellular globular domains (α2, or α3, or both) are smaller than HLA-G1/-G5 isoforms and are not associated with β*2*m (23). HLA-G1 to HLA-G4 are membrane-bound isoforms due to the presence of the transmembrane region encoded by exon 5 and a short cytoplasmic tail encoded by exon 6, which contains a stop codon. HLA-G5 and HLA-G6 are soluble isoforms due to the presence of intron 4, which contains a premature stop codon to prevent the translation of the transmembrane and cytoplasmic tail. HLA-G7 is a soluble isoform due to the presence of intron 2, which contains a premature stop codon and results in the expression of a soluble protein (18–20). All seven reported HLA-G isoforms contain the extracellular α1 domain.

In addition to the seven HLA-G monomers reported, the molecular structure of HLA-G is even more complex. A study on its crystal structure demonstrated that HLA-G can exist as a dimer with the intermolecular Cys42-Cys42 disulphide bond (24). *In vitro* and *in vivo* studies have shown that HLA-G dimers are observed for all isoforms except HLA-G3 (25). Moreover, β*2*m-associated and β*2*m-free dimers of HLA-G1 or HLA-G5 also exist (26–28). Dimer formation affects the specificity of receptor-HLA-G binding, as dimers exhibit a higher overall affinity to immunoglobulin-like transcript (ILT)2/4 receptors than monomers due to significant avidity effects (24, 28, 29).

Notably, unidentified HLA-G isoforms without an α1 domain were predicted based on RNA sequencing (RNA-seq), and several previously undescribed HLA-G isoforms have been identified in renal cancer samples (30). According to the nucleotide sequence of the *HLA-G* gene listed in the Ensembl database (ENST00000376828), this gene may possess a supplementary exon at the 5′-end, but this is absent from the sequence in the IMGT/HLA database. A novel HLA-G isoform named HLA-G1L was predicted by Tronik-Le Roux et al. (30); this isoform has five additional amino acids (MKTPR) located at the N-terminal end. Analysis of RNA-seq data indicates that some sequence reads may be initiated at exon 4, and thus could predict the existence of novel α1-deleted HLA-G isoforms that contain α2 and α3 domains or only the α3 domain. Other novel soluble HLA-G isoforms can be generated by the skipping of exon 6 coding for the transmembrane domain (30, 31). Lin et al. (32) indicated the existence of novel α1-deleted HLA-G isoforms containing intron 4 in 11.6% (44/379) of colorectal cancer lesions that exhibited negative staining with mAb 4H84 but that exhibited positive staining with mAb 5A6G7 (4H84^neg^5A6G7^pos^). Moreover, patients with 4H84^neg^5A6G7^pos^ HLA-G isoforms had a better survival than patients with 4H84^pos^5A6G7^neg^, and thus suggests a functional role for the novel α1-deleted HLA-G isoforms (31). However, the specific function of these novel HLA-G isoforms remains to be determined. The development of specific antibodies for these novel HLA-G isoforms is urgently needed and even inevitable (33).

## HLA-G-Mediated Immune Suppression

HLA-G expression was initially observed on cytotrophoblasts at the maternal-fetal interface (34), where HLA-G modulates the response of maternal immune cells that contribute to maintenance of tolerance to the fetus (35–37). HLA-G has a physiological tissue-restricted distribution property, as it is expressed by cytotrophoblasts (34), cornea (38), thymus (39), nail matrix (40), pancreatic islets (41), and erythroblasts (42). However, aberrant upregulated expression of HLA-G molecules has been detected in pathological conditions such as malignancies (43–45), infections and inflammatory diseases (14, 46–49), transplant grafts (50, 51), and autoimmune disorders (16, 52–54). In malignancies, aberrant HLA-G expression was preferentially detected in tumor tissues but was rarely detected in normal or adjacent non-tumorous tissues, which indicates that HLA-G might play a key role in tumor development (44).

**Figure 1:**
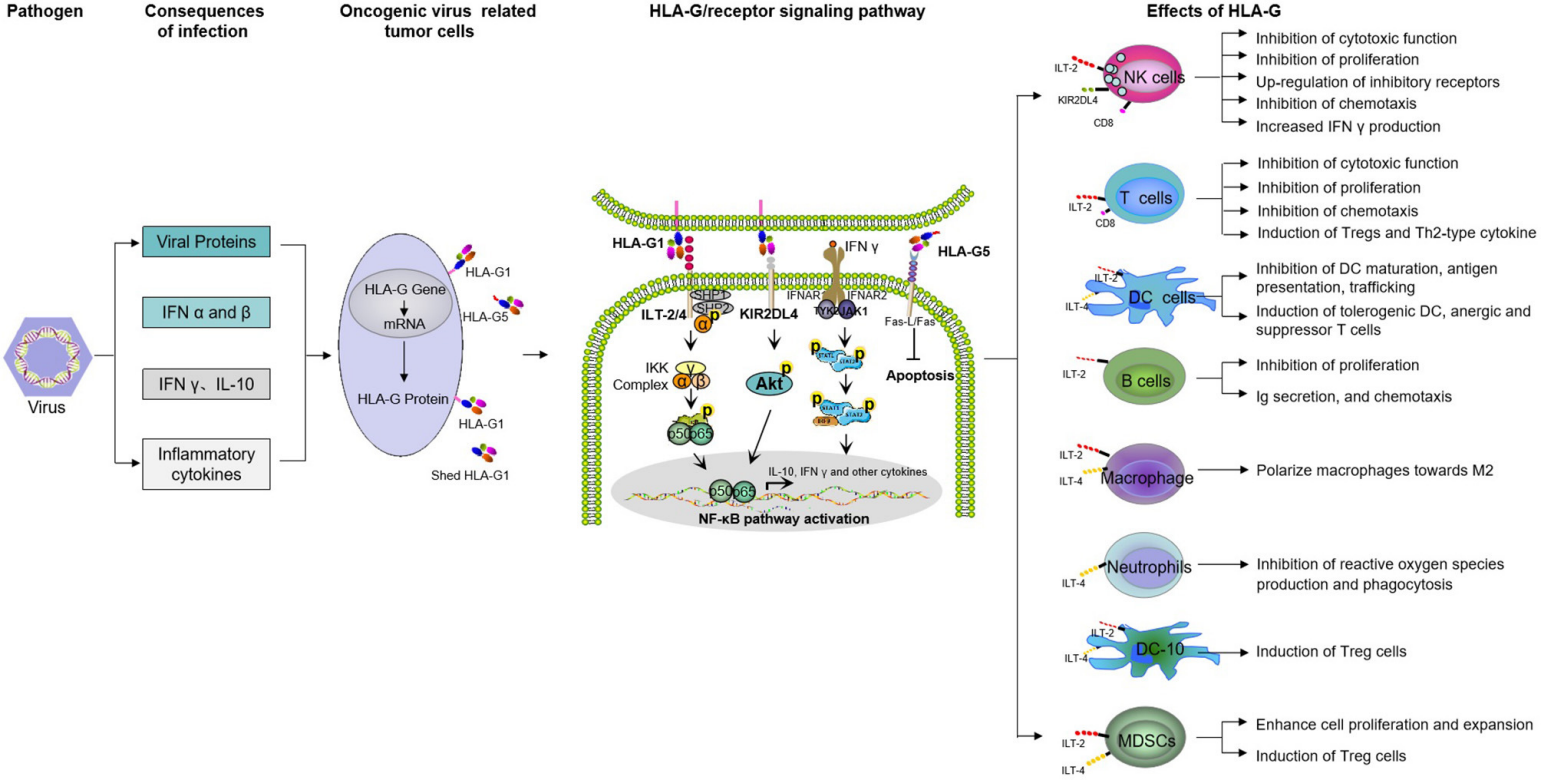
Mechanisms of both membrane-bound and soluble HLA-G-mediated immune suppression in tumor immune evasion. NK, natural killer cells; DC, dendritic cells; MDSC, myeloid-derived suppressor cells; Tregs, regulatory T cells; DC-10, IL-10-differentiated dendritic cells.

Functionally, HLA-G has comprehensive immunosuppressive properties exerted in multiple steps to weaken anti-tumor immune responses by acting on immune cells through its inhibitory receptors: ILT2(CD85j/LILRB1), ILT4(CD85d/LILRB2), and KIR2DL4(CD158d) (11, 12, 55–59) (Figure 1). HLA-G inhibits the cytolytic function of natural killer (NK) cells (60, 61), cytotoxic T lymphocyte (CTL)-mediated cytolysis (62), macrophage-mediated cytotoxicity (63), allo-proliferative response of CD4^+^ T cells (64, 65), maturation and function of dendritic cells (DCs) or B lymphocytes (66–69), stimulation of antigen-presenting cells (APCs) to secrete functional cytokines TGF-β and IL-10, and induction of apoptosis of CD8^+^ T cells and CD8^+^ NK cells (70, 71). In addition, HLA-G-receptor interactions could also exert long-term immunomodulatory effects by inducing immune suppressor/regulatory cells, such as regulatory T cells (Tregs) (72, 73), tolerogenic DCs (tDCs) (74, 75), mesenchymal stem cells (MSCs) (76), and myeloid-derived suppressor cells (MDSCs) (77, 78), among others. In addition to the interactions between HLA-G and its receptors, HLA-G-mediated immunosuppression by intercellular transfer mechanisms such as trogocytosis, exosomes, or tunneling nanotubes (TnTs) also represents another important complementary mechanism through which cancer cells escape destruction by the host immune system (11, 12, 79–81).

## HLA-G Polymorphisms in the Context of HPV Infections

To date, 69 alleles that encode 19 proteins have been discovered (IMGT/HLA Database, February 2020). Polymorphic sites along the *HLA-G* gene may change the affinity of gene-targeted sequences for transcriptional or post-transcriptional factors (82, 83). In particular, the *14bp Insertion/Deletion (Ins/Del)* (rs66554220) in the 3′UTR is associated with *HLA-G* alternative splicing and mRNA stability (84, 85). The +*3142C/G* (rs1063320) located 167 bp downstream from the *14bp Ins/Del* polymorphic site may be a target for HLA-G-specific miRNAs (86), which could directly downregulate HLA-G expression through post-transcriptional regulation (87, 88).

Accumulating evidence has supported the concept that HLA-G polymorphisms are genetic susceptibility and/or protection-relevant factors for cervical HPV infections and viral persistence (89–101). Many studies have primarily focused on polymorphisms in the 3′UTR of the *HLA-G* gene (89–95), while few have assessed its promoter region (96). Studies by Xu et al. (89, 90) showed that *HLA-G 14bp Ins* or +*3142G* alleles are risk factors for HPV infections, especially hrHPV infections, compared with the alleles found in healthy women and that these alleles affect the progression of HPV18-associated cervical lesions in Chinese women. A similar finding was reported in a study performed in Brazilian women from São Paulo, Brazil; this study showed that the *HLA-G 14bp Ins/*+*3142G* haplotype was related to increased risk of high-grade cervical lesions, especially in smokers (91). Inconsistent results were obtained in Italy (92) and Taiwan (93), where increased risk for squamous cell carcinoma (SCC) was found to be associated with the *14bp del* or +*3142C* alleles, especially in SCC patients infected with the HPV16 genotype (93). Moreover, some have focused on the association between *HLA-G* 3′UTR polymorphisms and HPV infection among HIV-positive women who have a higher risk of developing HPV co-infection. The combination of the +*3142CX (CC or CG)* and +*3187AA* genotypes conferred the highest risk of HPV-induced aneuploidy in cervical cells among Brazilian women with HIV/HPV co-infections (94).A SNP (rs1633038) in the 3′UTR of the *HLA-G* gene was significantly related to higher HPV clearance rates among African-Americans with HIV/HPV co-infection, but this association was not observed in Hispanics or European-Americans (95).

Further evidence for the role of genetic factors in HPV infections and the carcinogenic process was provided by studies that showed an association with specific HLA-G coding region polymorphisms (96–104). Among the Canadian population, the HLA-G^*^01:01:02 and HLA-G^*^01:03 alleles were found to be related to an increased risk of HPV16 infection and persistent infections (96), while the HLA-G^*^01:01:03 and HLA-G^*^01:01:05 alleles were identified as significant predictors of cumulative coinfections over the follow-up period (97). In the same cohort, the HLA-G^*^01:01:02, HLA-G^*^01:04:01 and HLA-G^*^01:06 alleles were related to high-grade cervical intraepithelial neoplasia (HG-CIN) (98). The HLA-G^*^01:01:02, HLA-G^*^01:06 and 3′UTR *14bp Ins* alleles were associated with disease progression from preinvasive to invasive cervical cancer among HPV-positive Canadian women (99). The homozygous HLA-G^*^01:04:01 genotype was related to a significantly decreased risk of HPV infection (98), and the heterozygotic form of the HLA-G^*^01:01:01 allele conferred significant protection against cancer (99). Among Brazilian women, the HLA-G^*^01:04/14bp Ins haplotype as well as HPV16 and HPV18 co-infection were preferentially related to HG-CIN, while the HLA-G^*^01:03 allele was related to protection against HPV-related cervical lesions (100). Among HPV-positive pregnant women in Brazil, a protective effect of the HLA-G^*^01:01:02 allele against the occurrence of CIN was observed in a cohort of HPV/HIV co-infected pregnant women (101). In a study that focused on the role of host factors in the vertical transmission of HPV infection from mother to offspring, the results showed that the HLA-G*01:01:01/01:04:01 genotype increased the risk of hrHPV infection in both cord blood and the infant's oral mucosa; moreover, the mother-child concordance of HLA-G*01:01:02/01:01:02 increased the risk of oral hrHPV infection both in the mother and offspring (102). In addition, a pilot analysis of HLA-G promoter methylation and HPV infection status showed no association between HLA-G methylation and HPV infections in healthy women (103, 104).

Overall, the discrepancy among these studies could be explained by differences in the study designs, ethnicity, sample sizes, and cancer types. Current data suggested that HLA-G gene polymorphisms (mainly located in the coding region or 3′UTR region) appear to be independent risk factors for HPV infection and cervical carcinogenesis, which supports the biological role of HLA-G molecules in shaping the tumor microenvironment (105).

## HLA-G Expression in Cervical Carcinogenesis

HLA-G expression may be induced after HPV infection, which leads to escape from host immunosurveillance. This evidence is derived from the results of a study that showed that HLA-G expression was significantly higher in CIN and cancer patients with HPV16/18 infections than in CIN patients without HPV infection (106). Several studies have investigated the relationship between HLA-G isoform expression and clinicopathologic features in patients with precancerous lesions and invasive cervical cancer (45, 90, 106–117).

Another study focused on HLA-G mRNA expression in cervical cancer in Korean patients using RT-PCR (15 normal tissues and 40 cervical cancer tissues) and found that high HLA-G mRNA expression was related to the early stages of cervical cancer (108). These results are consistent with the report by Rodriguez et al. (109), which showed upregulation of HLA-G protein expression in the early stages of cervical cancer in Colombian patients using immunohistochemistry (IHC) with mAb 4H84 (9 CIN III and 54 cervical cancer cases). Both studies supported a possible role for the HLA-G molecule in early cervical carcinogenesis (108, 109). The results of both studies further showed that Interleukin-10 (IL-10) expression was also significantly increased in cervical cancer tissues (108, 109), which supports a shift toward a Th2 cytokine microenvironment; this in turn may promote local immunosuppression by upregulating HLA-G expression (111, 118). Consistent with this, the results also revealed the inverse relationship among HLA-G expression levels and estimated numbers of tumor infiltrating lymphocytes (TILs) and CD57+ NK cells, which favors an escape from host anti-tumor activity (115). Moreover, three independent studies have reported evidence of a positive correlation between HLA-G expression and cervical carcinogenesis in a Chinese population (45, 106, 107). The results of all three studies indicated that HLA-G expression was negative in normal or adjacent non-tumorous tissues but was significantly increased along with CIN grade and cervical cancer metastasis. HLA-G expression may play an important role in determining the risk for cervical carcinogenesis. These clinical studies also analyzed clinicopathological parameters and demonstrated significant correlations between HLA-G expression and unfavorable prognosis, poor overall survival, and lymph node metastasis. However, inconsistent results were obtained in only two studies that showed that HLA-G expression was not related to cervical carcinogenesis (112, 113). Zhou et al. (112) described that in all normal epithelium, HLA-G expression was strong and uniform but was statistically down-regulated in CIN and SCC. Gonçalves et al. (113) reported that HLA-G was not expressed in any CIN or SCC. Futhermore, in experimental model of cervical cancer research, Real et al. (119) reported that low expression of HLA-G in Hela cell line (HPV18 infection). Thus, the role of HLA-G in malignancies has gained considerable clinical interest due to the possibility of exploiting it as a novel diagnostic/prognostic biomarker to identify cervical cancer and to monitor disease stage.

Additionally, three studies focused on soluble HLA-G (sHLA-G) isoform expression using different detection technologies (107, 110, 114). Guimarães et al. (110) analyzed sHLA-G expression in cervical cancer tissues from Brazilian patients using IHC with the specific mAb 5A6G7 (27 with metastasis and 52 without metastasis). Low expression of sHLA-G isoforms was detected in all HPV-positive tissues, and the sHLA-G expression level was similar in both groups (110). Zheng et al. (107) investigated the sHLA-G expression level in the plasma of patients with cervical lesions using ELISA kit (sHLA-G, Exbio) with mAb MEM-G/9 (20 normal cervical tissues, 15 CIN I, 22 CIN II, 35 CIN III, and 80 cervical cancer tissues). sHLA-G expression levels in the plasma were significantly increased in CIN II-III and SCC patients, and their expression levels were also associated with differentiation and metastasis. Therefore, sHLA-G molecules may have significance in early cervical cancer screening (107). However, inconsistent results obtained in the Netherlands (366 cervical cancer) using ELISA kit (sHLA-G, Exbio) reported that sHLA-G levels were not associated with clinicopathological parameters or survival (114).

Overall, the discrepancies in these studies that examined HLA-G expression in cervical cancer patients are partly due to tumor heterogeneity (31). In the future, there will be a need for additional studies to obtain deeper insight into the association between HLA-G expression levels and advanced cervical cancer.

## HLA-G as a Novel Target for Immunotherapies

Cervical cancer accounts for 6.6% of all female cancers and is thus a major global health challenge, as ~90% of cervical cancer deaths occur in less developed countries (1). High-risk HPV causes almost all invasive cervical cancers, and therefore, HPV screening and vaccination are needed to improve cervical cancer control (2). Despite significant advances in effective screening and preventive vaccination during the past decade, substantial regional and global disparities in the prognosis of cervical cancer patients still exist (120). Unfortunately, ~30% of patients experience recurrence and metastasis after primary treatment, with an expected 5-year survival of <10%. Few effective therapeutic strategies have been developed that specifically target recurrent or metastatic cervical cancer, particularly advanced-stage disease. Thus, novel therapeutic strategies, such as immunotherapy, are urgently needed in clinical settings (121, 122).

In recent years, an improved understanding of the molecular mechanisms of the interactions between HPV-associated cervical cancer and the host immune responses has driven the exploration of immunotherapy as one of the new therapeutics targeting immune checkpoints (123). HLA-G has comprehensive immunosuppressive properties that are exerted in multiple steps to weaken the anti-tumor immune responses by acting on immune cells through its inhibitory receptors. Fortunately, HLA-G expression can be downregulated through RNA interference or antibody blockade, which can allow recovery of the functions of immune effectors and prevent tumor reoccurrence. Thus, HLA-G could serve as a novel immune checkpoint molecule and play a key role in novel immunotherapy approaches that offer a promising perspective for tumor progression and advanced- stage cervical cancer.

It has been confirmed that miR-148a negatively regulates HLA-G expression by binding to the 3′UTR of the *HLA-G* gene (88). The long non-coding RNA HOX transcript antisense RNA (HOTAIR) may also serve as a competing endogenous RNAs (ceRNAs) to regulate HLA-G expression by sponging miR-148a in cervical cancer cells (116). Targeting the HOTAIR-miR-148a-HLA-G axis or HLA-G-specific miRs could represent a novel therapeutic strategy in cervical cancer. Intra-tumor heterogeneity of checkpoint molecule expression in cervical cancer is related to a poor chemo/radio-therapy response, lymph node metastasis and tumor recurrence. HLA-G has been identified as a cervical cancer stem cell (CCSC)-specific marker, and targeting HLA-G and its related signaling pathways may offer a novel strategy for CCSC-targeted therapy (124). Moreover, the HLA-G/ILTs axis has been recently recognized as a new immune checkpoint in addition to other immune checkpoints such as programmed cell death 1 (PD-1)/PD-L1 and cytotoxic T lymphocyte-associated protein 4 (CTLA-4)/B7 (125). Different responses to checkpoint inhibitor therapy could be a consequence of heterogeneous intra- and inter-tumor expression of different types of checkpoint molecules, although data on the expression status of HLA-G, CTLA-4 and PD-L1 in cancers are rather limited (13, 125, 126). PD-1 is a major immunotherapeutic checkpoint target in various cancer types, but until now, few data have been available on the clinical efficacy of blocking this checkpoint protein in cervical cancer (2, 126). The expression of PD-1 was found to be heterogeneous in tumors and could be co-expressed with the immune checkpoint protein HLA-G (127). A recent study focused on tumor-infiltrating CD8+ T cells that express the HLA-G receptor ILT2 in renal-cell carcinoma (RCC), and the results emphasize the potential of therapeutically targeting the HLA-G/ILT2 checkpoint in HLA-G+ tumors (127). Overexpression of the immune checkpoint HLA-G molecule by tumor cells profoundly affects tumor-specific T cell immunity in the cancer microenvironment. In this regard, targeting multiple checkpoints, especially potential antagonists of the HLA-G/ILT-2/4 pathway, is urgently needed to target the entire tumor.

## Conclusions

Considering the above studies that were reviewed, we propose that *HLA-G* gene polymorphisms have an impact on the immune response and likely determine those in specific populations who are at higher risk for cervical HPV infections and viral persistence. Aberrant HLA-G expression in cervical lesions could generate inhibitory signals in the cancer microenvironment, which would ultimately help tumor cells escape from immunosurveillance and reshape tumor progression and metastasis. The checkpoint molecule HLA-G with immune tolerance contribute to cervical carcinogenesis, but HLA-G could also represent a good immunotherapeutic target for cervical cancer treatment.

## Author Contributions

All authors listed have made a substantial, direct and intellectual contribution to the work, and approved it for publication.

## Conflict of Interest

The authors declare that the research was conducted in the absence of any commercial or financial relationships that could be construed as a potential conflict of interest.
